# Metabolic response to three different diets in lean cats and cats predisposed to overweight

**DOI:** 10.1186/s12917-017-1107-3

**Published:** 2017-06-19

**Authors:** Claudia Keller, Annette Liesegang, Diana Frey, Brigitta Wichert

**Affiliations:** 1 Institute of Animal Nutrition, University of Zurich, Winterthurerstr, 270, CH-8057 Zurich, Switzerland; 2 Department of Rheumatology, University Hospital Zurich, Zurich, Switzerland

**Keywords:** Glucose, Insulin, Protein, Fat, Carbohydrate

## Abstract

**Background:**

The existence of a genetic predisposition to obesity is commonly recognized in humans and rodents. Recently, a link between genetics and overweight was shown in cats. The goal of this study was to identify the effect of diet composition on plasma levels of glucose, insulin, free fatty acids and triglycerides in cats receiving different diets (high-carbohydrate, high-fat and high-protein diets).

**Results:**

Insulin and leptin concentrations were significantly correlated with phenotype. Insulin levels were lower, whereas leptin levels were higher in cats predisposed to overweight. The other blood parameters were not correlated with phenotype. Intake of the high-carbohydrate diet resulted in higher insulin concentrations compared with the two other diets. Insulin levels were within the values described for non-obese cats in previous studies.

**Conclusions:**

There was no difference in metabolic response between the two groups*.* As the high-carbohydrate diet led to the highest insulin blood concentrations, it might be useful to avoid such diets in cats predisposed to overweight. In addition, even cats with genetically linked obesity can regain insulin sensitivity after weight loss.

## Background

Overweight in humans is one of the major health risks worldwide [1]. It is defined as abnormal or excessive accumulation of fat [1]. Pets, particularly dogs and cats, share the same environment as humans, in which they lack exercise and have unlimited access to a high-calorie diet [2], which are the reasons why overweight and obesity are among the major health problems in these species [3, 4]. In humans overweight is a main risk factor for a number of secondary disorders, such as type 2 diabetes mellitus, cardiovascular diseases, orthopaedic problems, urogenital disorders, neoplasia and anaesthetic complications [5, 6]. Similarly, overweight is a main risk factor for secondary disorders in cats. These include among others, diabetes mellitus, orthopaedic problems and anaesthetic complications [5]. The secondary disorders lead to reduced life span and quality of life [5, 6]. Feline diabetes, which is associated with overweight, is very similar to human type 2 diabetes [2].

Risk factors for obesity in humans include the following: excessive intake of highly palatable and energy-rich food, a diet that does not meet all nutrient requirements and lack of physical activity [7]. Additionally, genetic background is an important risk factor for obesity in humans and laboratory animals as well as in cats [7–9].

Excessive body weight caused by excessive body fat content leads to reduced insulin sensitivity and can later lead to hyperglycaemia [10, 11]. Both symptoms are warning signals for type 2 diabetes [11, 12]. Reduced insulin sensitivity due to an increase in body fat is reversible with weight loss [3, 13] in humans and cats. It is therefore important to prevent humans and cats from being overweight and to reduce existing excessive body weight. It is known that obese cats are insulin resistant, but weight loss normalizes this insulin resistance [12, 14]*.*

It is hypothesized that overweight is caused by several interacting genes and the environment [7]. The genetic background of obesity in cats has not been examined as thoroughly as in humans and mice [7, 15–17]. Häring et al. [9] found inheritance of obesity in an experimental cat population. In a later study, Wichert et al. [18] found that cats predisposed to overweight (*po*) show lower energy requirements and higher food intake even in ideal body condition. The authors identified one major gene model with a polygenic component linked to obesity. The analysis identified genomic regions associated with overweight [19]. In another study, a genetic analysis identified a missense mutation in the coding sequence of *MC4R* (*MC4R:C.92 > T*) related to diabetes mellitus in obese cats [20]. The same missense mutation is also involved in the development of human obesity and type 2 diabetes mellitus [21]. The cats used in the present study originated from the population that was phenotyped by Häring et al. [9].

Hoenig et al. also reported a decreased glucose effectiveness in obese cats [12]*.*

The influence of macronutrient composition on energy metabolism and satiety is controversial in cats as well as in humans. Scarlett et al. [3] identified a high carbohydrate diet to be a risk factor for overweight in cats, as opposed to a high-fat canned diet. In contrast to this, Backus et al. [22] observed a negative correlation between carbohydrate content and body weight while a high-fat diet was a risk factor for overweight. It is also known that carbohydrate sources influence postprandial glucose and insulin levels [23]. In studies on the influence of nutrient components on metabolic reactions, different combinations of nutrient components were used and they are therefore not fully comparable. In other studies, protein rich diets caused higher metabolic rates than low protein diets [24] and conserved fat-free mass during weight loss [25, 26] in overweight cats. There is also evidence in humans that high-protein diets cause higher weight loss and fat mass loss than high-fat or high carbohydrate diets [24, 27]. The most important reasons for this beneficial effect are earlier and quicker satiety and lower energy intake after high-protein meals [27].

The goal of the present study was to identify whether a genetically caused predisposition to overweight influenced the plasma levels of glucose, insulin, leptin, free fatty acids and triglycerides in cats at ideal body condition when they were fed three different diets (high carbohydrate, high fat and high protein). It was hypothesized that an inherited predisposition to overweight influences plasma levels of these blood parameters in ideal body condition. If the influences of different genetic predispositions on plasma levels of these blood parameters were known, it might be easier to know which diets to feed in order to prevent obesity and the development of diabetes mellitus in cats.

## Methods

### Animals

Thirteen clinically healthy, intact adult (four to five years old) male European short-hair cats from the Institute’s owned feline colony. The cats were divided into two groups (six *po* and seven lean (*l)* cats) based on classification by phenotype. The classification was determined by BCS [9] at the age of eight months. To reach ideal body condition, the cats of group *po* underwent a weight loss programme. During this weight loss programme, the cats were fed commercial canned food. At least four weeks before the beginning of the trial, they were fed to weight constancy*.* All cats had an ideal BCS of 5–5.5/9 [28] for at least 4 weeks before the beginning of the experimental trial. Body composition was measured using dual-energy X-ray absorptiometry (DXA) at the beginning and at the end of the study. Ethical approval for the experiments was obtained from the local Ethics Committee for Animal Experiments (Veterinaeramt des Kantons Zuerichs; licence number 83/2012).

### Experimental design

Three non-commercial experimental diets were prepared: one with high carbohydrate content (HCH), one with high fat (HF), and another with high protein content (HP). The diets were fed in an order determined by a Latin square design. The diets contained beef, pork liver, lard and cooked white rice (only in HCH). The metabolizable energy (ME) and crude nutrient content are given in Table 1. The diets were composed according to adult feline requirements [29], with no nutrient deficiencies. Macro and trace elements as well as vitamins and taurine were added individually to each experimental diet.

**Table 1:** Crude nutrient content of the diets

	Crude protein (% DM)	Crude fat (% DM)	Crude ash (% DM)	ME (kJ/g dry matter)
HCH	32.5	24.2	1.7	20.0
HF	29.9	62.7	1.2	26.5
HP	71.3	28.5	0.2	21.9

*HCH* high carbohydrate, *HF* high fat, *HP* high protein

Crude protein, crude fat and crude ash in percentage of dry matter (% DM), metabolizable energy (ME) in kJ per g dry matter [31]

All cats were fed for maintenance of body weight (BW). The leftovers were weighed after each meal, and food intake was adjusted to BW. After each feeding phase, the cats had a wash-out period of 14 days during which they were fed with adult canned food (dry matter (DM) 19%, crude protein 41% DM, crude fat 24% DM, crude fibre 2% DM, crude ash 2.5% DM). The cats were fed four times a day, at 8:30, 11:00, 13:30, and 16:00. On blood sampling days only, the meal at 11:00 was cancelled. The cats were fed separately for 15 min each. Each cat was weighed every morning before the first feeding. If its BW changed, the amount of food was adjusted in steps of 0.1 MJ ME per day. BCS was assessed at the beginning and the end of the feeding periods as well as between the feeding periods once a month.

**Fig. 1:**
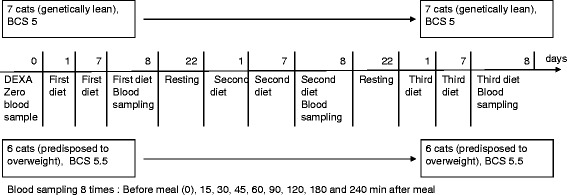
Experimental design. Two groups of cats cats, one lean *(l,* 7 cats*)* and one with predisposition to overweight (*po*, 6 cats). Three diets (high-carbohydrate (HCH), high-protein (HP) and high-fat (HF)), each fed for 7 days in an order determined by a Latin square. At day 8: blood sampling before (0) and at eight timepoints after (15, 30, 45, 60, 90, 120, 180 and 240 min) a meal of one of the three test diets. Fourteen-day washout period

After a fasting period of 16 h, a blood sample at time zero was taken. Then, the cats were fed and additional blood samples were taken as shown in the time table (Fig. 1). Blood samples were analysed for glucose, insulin, triglyceride, free fatty acid and leptin concentrations.

### Analyses

For DXA measurements the cats were sedated with 0.075 mg/kg BW Medetomidine (Dorbene® ad us. vet., Dr. E. Graeub AG, Bern, Switzerland) and 0.25 mg/kg BW Butorphanol (Morphasol-4 ad us. vet., Dr. E. Graeub AG, Bern, Switzerland) intramuscularly in a mixed syringe. The cats were placed in a prone position [9] and scanned using an infant whole-body scanner. The measurements were performed with a Hologic Discovery A Densitometer (S/N 85508; Hologic Inc., Bedford, Massachusetts, USA) and the QDR system software, version 13.3.3 (Hologic Inc., Bedford, Massachusetts, USA) [30].

Each diet was homogenized and random samples were collected. The samples were analysed by proximate analysis (Table 1). The content of ME was estimated based on the results of the proximate analysis and the formulas of Kienzle et al. [31].

For blood collection, the cats were sedated with 10 mg/kg BW ketamine (Ketanarkon 100 ad. us. vet., Streuli Pharma AG, Uznach, Switzerland) and 0.2 mg/kg BW midazolam (Dormicum ®, Roche Pharma AG, Reinach, Switzerland) intramuscularly in a mixed syringe. A catheter (20 G) was used if more than one blood sample was taken from the vena cephalica. The sedation was maintained with propofol (Fresenius Kabi AG, Bad Homburg von der Höhe, Germany) as needed. It is known that this medication has only an insignificant effect on the measured blood parameters [32–34].

Blood glucose was analysed immediately after blood sampling using a portable glucometer (Ascensia Elite™, Bayer Corporation, Mishawaka, IN, USA) [35]. Blood samples were centrifuged and the plasma was stored at −80 °C until further analysis. Plasma insulin levels were determined by an enzyme-linked immunosorbent assay (ELISA; Feline Insulin ELISA, Mercodia, Uppsala, Sweden). Strage et al. [36] validated this test kit for insulin measurement in cats and found intra- and inter-assay coefficients of variation of 2.0–4.2% and 7.6–14%, respectively. For the analysis of triglycerides (TRIG Diatools, Villmergen, Switzerland) and free fatty acids (NEFA-HR(2), Wako, Neuss, Germany) colourimetric measurements were performed with the help of a Cobas Mira® (Hoffmann-La Roche, Basel, Switzerland). Leptin content was measured using RIA (Multi-Species Leptin RIA Kit, Millipore, Missouri, USA). This test kit was developed to measure leptin in many species, and has been validated for use in cats [37]. The intra- and interassay coefficients of variation were 2.8–3.6% and 6.5–8.7%, respectively.

Insulin sensitivity was determined by homeostasis model assessment (HOMA) [38]. This measure is the product of insulin and glucose divided by 22.5. The HOMA index was developed to measure human insulin sensitivity and was validated for cats by Appleton et al. [39].

The results are presented as the mean ± standard error (SE). A multivariate analysis of variance (MANOVA) for repeated measurements was performed with group (lean or predisposed to overweight) as a cofactor included in the model. The impact of correlated factors was tested by linear regression analysis with help of SPSS® Statistics 20.0 (IBM Corporation, New York, United States). A t-test was performed to compare pairs of groups with help of Microsoft Office Excel 2013 (Microsoft Corporation, Redmond, WA, United States).

## Results

During the whole study, genetically lean cats had a mean BCS of 4.9 (± 0.01) and cats predisposed to overweight had a BCS of 5.1 (± 0.02). Mean BCS change during the whole study was 2.6%, and mean weight change during a single experimental period was 0.72% for all cats. The greatest change in weight was measured with diet HF (1.25%), and the smallest with diet HP (0.28%).

At the beginning of the experiment, the cats of group *po* had a mean fat mass percentage of 8.5% (± 1.28) and a fat-free mass percentage (including bone) of 91.7% (± 2.06), while group *l* had a fat mass percentage of 6.0% (± 0.72) and a fat-free mass percentage of 91.5% (± 0.69). The BCS at this date was 5.24 (± 0.03) for group *po* and 4.99 (± 0.03) for group *l*. At the end of the experiment, group *po* had 4.0% (± 0.72) body fat mass percentage and 93.4% (± 0.69) fat-free mass percentage, while group *l* had a 5.2% (± 1.18) fat mass percentage and a 92.0% (± 1.17) fat-free mass percentage. At this date, the BCS was 5.00 (± 0.05) for group *po* and 4.97 (± 0.04) for group *l*.

For the HCH diet, the mean daily energy intake was 333.3 (SE 15.7) kJ ME per BW^0.67^ in group *po* and 379.2 (SE 13.8) kJ ME per BW^0.67^ in group *l*. For the HF diet, the mean daily intake was 352.7 (SE 14.6) kJ ME per BW^0.67^ in group *po* and 363.9 (SE 10.9) kJ ME per BW^0.67^ in group *l*. Finally, for the HP diet, the mean daily intake was 290.2 (SE 13.9) kJ ME per BW^0.67^ in group *po*, and in group *l* it was 392.0 (SE 11.15) kJ ME per BW^0.67^.

**Fig. 2:**
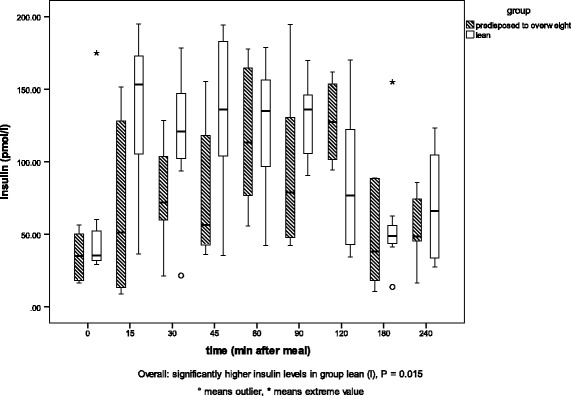
Plasma insulin concentrations in the two groups after a high-carbohydrate (HCH) meal. Plasma insulin concentrations [pmol/l] in the two groups of cats (lean (*l*) and predisposed to overweight (*po*)) before (0) and at eight timepoints between 15 and 240 min after anHCH meal

**Fig. 3:**
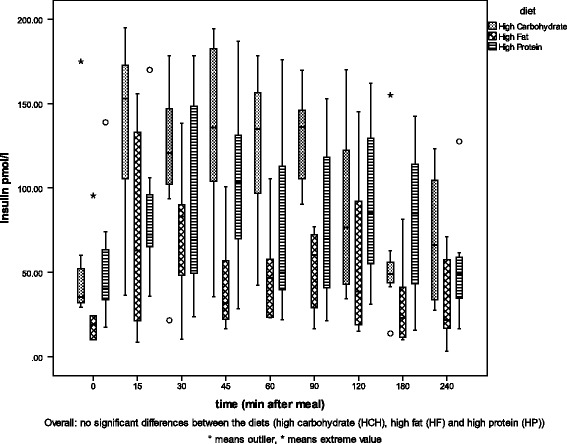
Plasma insulin concentrations of the lean group (*l*). Plasma insulin concentrations [pmol/l] of group *l* before (0) and eight times between 15 and 240 min after a meal of each of the three test diets (high carbohydrate (HCH), high fat (HF) or high protein (HP))

**Fig. 4:**
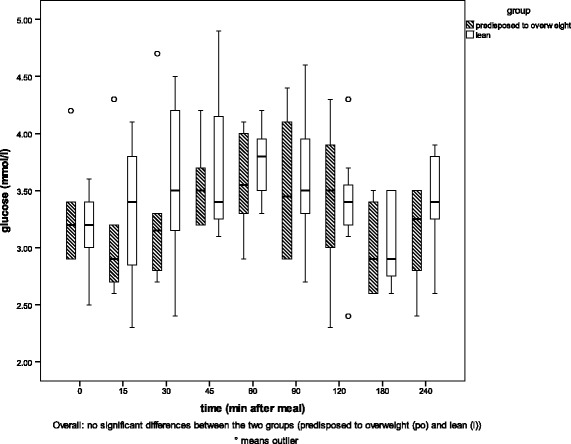
Plasma glucose concentrations in the two groups of cats (lean (*l*) and predisposed to overweight (*po*)). Plasma glucose concentrations [ng/l] in the two groups of cats (lean (*l*) and predisposed to overweight (*po*)) before (0) and at eight timepoints between 15 and 240 min after a meal of high carbohydrate (HCH) diet

**Fig. 5:**
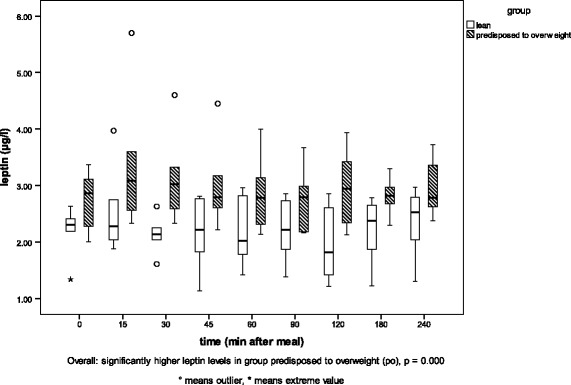
Plasma leptin concentrations in the two groups of cats. Plasma leptin concentrations [μg/l] in the two groups of cats (lean (*l*) and predisposed to overweight (*po*)) before (0) and at eight timepoints between 15 and 240 min after a high-fat (HF) meal

The results of the regression analysis are given in Table 2. Between the two genetic phenotypes (*l* and *po*), no differences were detected in glucose or triglyceride concentrations in blood plasma, but insulin and leptin concentrations were significantly correlated with phenotype (*p* = 0.006, *p* = 0 0.01). Group *l* cats showed higher insulin concentrations than group *po* cats. Cats predisposed to overweight showed higher leptin concentrations than lean cats. Diet was significantly positively correlated with glucose, insulin, leptin and triglyceride levels (*p* < 0.05) but only weakly positively correlated with free fatty acids (*p* = 0.056). Time after feeding was significantly positively correlated with blood glucose, insulin and free fatty acid values (*p* < 0.002). Plasma concentrations increased after a meal. Food intake was positively correlated with leptin and triglycerides (*p* < 0.05). The detailed relationships of glucose, insulin, leptin and triglyceride levels with phenotype and diet are shown in Figs. 2, 3, 4, and 5.

**Table 2:** Results of the regression analysis

Regression coefficients	Individual	Phenotype	Diet	Time after meal	Food intake	r
Glucose	0.024*	0.298	0.014*	<0.0001*	0.907	0.294
Insulin	0.258	0.006*	<0.0001*	0.001*	0.503	0.417
Leptin	0.003*	0.01*	<0.0001*	0.323	0.006*	0.776
Free fatty acids	0.111	0.056+	0.047*	0.309	0.485	0.177
Triglycerides	0.102	0.715	0.075+	<0.0001*	0.047*	0.447

Factors influencing one or more of the blood parameters (glucose, insulin, leptin, free fatty acids and triglycerides), *p*-values. * significant correlation (≤0.05), + weak correlation (> 0.05)

Insulin sensitivity as determined by HOMA [38] showed no significant differences between the two groups (*l* and *po). P*-values were as follows for all diets: HCH (*p* = 0.18), HF (*p* = 0.18) and HP (*p* = 0.09).

In both groups feeding the HCH diet resulted in higher insulin concentrations than the other diets (Figs. 2, 3), although it was more pronounced in group *l* (*p* = 0.015) than in group *po*. On the HP diet, higher insulin concentrations were measured in group *l* than in group *po* (*p* < 0.0001) and on the HF diet, group *l* had a tendency toward higher concentrations than in group *po* (*p* = 0.066).

For all diets, the glucose plasma concentrations were not significantly different between the two groups (example after the HCH diet was fed - Fig. 4).

Plasma leptin concentrations are shown in Fig. 5. Cats in group *po* showed significantly higher leptin levels than cats in group *l* after an HF meal.

After intake of the HF diet, the highest increases in plasma triglyceride and free fatty acid values were seen 15 min after the meal. Intake of the HP diet led to significantly higher free fatty acid concentrations in group *po* than in group *l* (*p* = 0.007).

## Discussion

The presented study is the first to analyse the metabolic response to different diets in healthy, normal-weight, sexually intact male cats with or without predisposition to overweight. The influence of neutering on body weight and metabolic response has been shown in several other studies [40–43]. In the present study we used intact male cats specifically to exclude the influence of neutering.

Although all cats had an ideal BCS between 5 and 5.5, BCS in group *po* was significantly higher than in group *l*. However, body fat content measured by DXA did not differ between the two groups. BCS is a subjective and semi-quantitative method [5, 28], whereas DXA is an objective measurement. It can be speculated that the BCS of cats predisposed to overweight was overestimated because the abdominal fat (skin) pad, which leads to higher BCS, was misinterpreted and probably consisted of skin only. The measured body fat percentages were low in comparison to other studies with cats [44]. The machine’s lower limit for body fat measurement was approximately 4% [18]. Measured body fat percentages depend on position and machine type [45]. The measured body fat percentages are comparable with other measured data in our cat population [18, 46], which is highly important given the differences in the body fat content of the investigated cats in different phases of life.

The concentrations of some blood parameters such as insulin, glucose and free fatty acids change during body weight loss or body weight gain over time due to physiological reactions during the intake of macronutrients [12, 47]. Because of the four weeks maintenance of body weight before the experiment, the influence of food restriction on food intake and energy expenditure as well as metabolic response in the present study can be neglected. Wichert et al. [18] showed in an earlier study that energy requirements calculated per kg BW^0.67^ were not significant different in the cats (*l* and *po*) used in the present study*.*

Interestingly, group *l* had higher insulin blood concentrations than group *po*, although no differences in body weight or body fat were measured. Because all cats showed normal glucose concentrations, it can be speculated that the maintenance of glucose concentrations is functional. One explanation for the relatively low insulin concentrations in group *po* in combination with normal glucose concentrations is a higher degree of insulin sensitivity, which means that less insulin is used for the control of normal glucose concentrations [48]. Another explanation could be the hepatic glucose production, which is still functioning [12]. However, insulin sensitivity as measured by HOMA [38] showed no significant differences between groups or diets during the whole experiment. The cats of the present study were measured earlier by Häring et al. [46], but the cats predisposed to overweight were overweight during the previous study (BCS >6) [39]. In that study, the cats of group *po* showed impaired insulin sensitivity in the glucose tolerance test, and the calculated HOMA indexes were higher than in the present study but nevertheless within the reference range [39]. From this observation, it can be speculated that if cats of the overweight phenotypic trait lose weight, their insulin sensitivity is regulated to a normal status and reaches the normal range again. This “normalizing” effect of weight loss on insulin sensitivity, already described in several publications on cats [12, 13], is comparable to the reversal of insulin resistance by weight loss as described in humans [13]. The overweight-predisposed cats’ insulin regulation in this study responded physiologically.

Another explanation for the relatively low insulin concentrations in group *po* could be lower insulin production by beta cells in the pancreas. Thus, glucose levels could be maintained by other compensatory mechanisms such as glucagon [49]. As glucagon was not measured in the present study, this question cannot be answered here. The higher the glucose load, the higher the insulin flow necessary to maintain the glucose levels within the normal range [50], but in our study there was no difference in food intake between the two groups. In the present study, at least a tendency to higher insulin concentrations in group *l* was observed. Calculating the food intake per kilogram body weight, group *l* had a significantly higher food intake of HCH and HP diet compared to group *po*. Therefore, this finding seems to explain best the higher insulin concentrations in group *l*. One reason for the lower energy requirement of the cats predisposed to overweight could be lower energy expenditure, which was assumed for these cats earlier by Wichert et al. [18]. In the present study, the cats were fed to maintain ideal body weight and to reach the requirement. For all nutrients, *po* cats have similar metabolic responses to *l* cats, except that they still have lower energy requirements. One of the major differences from other studies was the individual feeding regimen, as in other studies a determined amount of food was used for all cats. The different results could be explained by the different feeding system. The assumed phenotypic trait of the cats in the present study does not seem to affect triglyceride levels. In our study, the triglyceride concentrations are higher with the HF diet, which is consistent with the literature [47]. Thiess et al. [47] showed higher triglyceride levels and reduced insulin response due to a high-fat diet. In addition, Wei et al. [24] measured higher triglyceride concentrations when feeding a high-protein diet than a moderate-protein diet, but the carbohydrate content of this high-protein diet was higher than in our experiments.

High-protein diets are postulated to be beneficial in promoting weight loss and better glycaemic control with normalized insulin levels in obese humans, who have been hyperinsulinaemic before [27, 28, 51]. Overall, there is some evidence that the beneficial influence of high-protein diets for weight loss and glycaemic control is similar in humans and cats. However, the results in the literature concerning cats are inconsistent. A study on cats [52] described a tendency toward higher insulin levels on a high-protein diet than on a high-carbohydrate or high-fat diet. In contrast, Backus et al. [22] showed the highest insulin concentrations when feeding a high-fat diet, and Hewson-Hughes et al. [53] showed higher insulin concentrations with a high-starch diet than with moderate-or low-starch diets in lean, healthy cats. In the present study, as in the study of Hewson-Hughes et al. [53], the highest insulin concentrations were measured with the HCH diet. It is known, that carbohydrate source also has a great influence on glucose and insulin response [23], and white rice is a very high-glycaemic carbohydrate source [23]. The HP diet produced lower mean insulin concentrations than the other 2 diets (HCH and HF) fed in the present study. Therefore, further studies are needed to determine the influence of macronutrients on glycaemic control in cats.

The present study revealed higher levels of leptin with the HCH diet than with the two other diets. According to the literature, we had expected to find increased leptin levels with a high-fat diet [47]. In contrast to Thiess et al. [47], who showed minimally higher leptin values with a high-fat diet, our study showed higher leptin levels with the HCH diet. However, Thiess et al., [47] did not determine the cats’ BCS and it is unclear whether the cats gained weight during the study. As shown by Backus et al. [22], leptin was not influenced by dietary fat content, but by body fat content. Since the DXA measurements showed no difference in the body fat content of the cats, the higher leptin levels with the HCH diet in the present study cannot be explained. Thus, the reason for the differences in the leptin concentrations in the present study remains unclear.

## Conclusion

To improve cats’ health, it is important to keep their weight within the normal range. This is more important for cats predisposed to overweight. The aim of the present study was to investigate whether cats predisposed to overweight react differently to diets with various macronutrient compositions (HCH, HP, HF) compared with lean cats. In the present study, no different metabolic response was measured between the predisposed to overweight and lean cats of the investigated cat colony. Only small differences in insulin levels could be shown. However, normal insulin sensitivity in the lean-state *po* cats was measured. It is still unclear why the *po* cats show same insulin sensitivity with lower plasma insulin concentrations. Thus, cats from our cat population, that return from overweight to normal weight have a minimized risk for insulin resistance and show normal insulin sensitivity, even if their insulin sensitivity was slightly decreased when they were overweight.

Due to these results in the present study, no differences in metabolic response in the measured blood parameters between cats with and without predisposition to overweight could be shown*.* The present data provide the first indications of beneficial effects on insulin sensitivity from avoiding high-carbohydrate diets with a high-glycaemic index, especially in cats with predisposition to overweight. As known from the literature, a high-carbohydrate diet with high-glycaemic carbohydrate sources increases the risk of weight gain. The results of the present study show that a high-protein diet and normal body weight could be advantageous for cats, consistent with its ability to prevent obesity and type 2 diabetes mellitus. This is another important hint that cats could be a useful model for obesity and type 2 diabetes development or prevention in human beings.

## Abbreviations

BCS: Body condition score
BW: Body weight
DXA: Dual-energy X-ray absorptiometry
HCH: High carbohydrate
HF: High fat
HOMA: Homeostasis model assessment
HP: High protein
l: Lean
ME: Metabolizable energy
po: Predisposed to overweight
SE: Standard error
